# Glucocorticoid receptor and nuclear factor kappa-b affect three-dimensional chromatin organization

**DOI:** 10.1186/s13059-015-0832-9

**Published:** 2015-12-01

**Authors:** Tatyana Kuznetsova, Shuang-Yin Wang, Nagesha A. Rao, Amit Mandoli, Joost H. A. Martens, Nils Rother, Aafke Aartse, Laszlo Groh, Eva M. Janssen-Megens, Guoliang Li, Yijun Ruan, Colin Logie, Hendrik G. Stunnenberg

**Affiliations:** Department of Molecular Biology, Faculty of Science Nijmegen, Radboud University, Nijmegen, The Netherlands; National Key Laboratory of Crop Genetic Improvement, College of Informatics, Huazhong Agricultural University, Wuhan, China; The Jackson Laboratory for Genomic Medicine, and Department of Genetic and Development Biology, University of Connecticut, 400 Farmington Ave., Farmington, CT 06030 USA

**Keywords:** ChIA-PET, Chromosome conformation capture, Enhancer, GR, Long-range interaction, NFκB, P300, Transcription regulation

## Abstract

**Background:**

The impact of signal-dependent transcription factors, such as glucocorticoid receptor and nuclear factor kappa-b, on the three-dimensional organization of chromatin remains a topic of discussion. The possible scenarios range from remodeling of higher order chromatin architecture by activated transcription factors to recruitment of activated transcription factors to pre-established long-range interactions.

**Results:**

Using circular chromosome conformation capture coupled with next generation sequencing and high-resolution chromatin interaction analysis by paired-end tag sequencing of P300, we observed agonist-induced changes in long-range chromatin interactions, and uncovered interconnected enhancer–enhancer hubs spanning up to one megabase. The vast majority of activated glucocorticoid receptor and nuclear factor kappa-b appeared to join pre-existing P300 enhancer hubs without affecting the chromatin conformation. In contrast, binding of the activated transcription factors to loci with their consensus response elements led to the increased formation of an active epigenetic state of enhancers and a significant increase in long-range interactions within pre-existing enhancer networks. De novo enhancers or ligand-responsive enhancer hubs preferentially interacted with ligand-induced genes.

**Conclusions:**

We demonstrate that, at a subset of genomic loci, ligand-mediated induction leads to active enhancer formation and an increase in long-range interactions, facilitating efficient regulation of target genes. Therefore, our data suggest an active role of signal-dependent transcription factors in chromatin and long-range interaction remodeling.

**Electronic supplementary material:**

The online version of this article (doi:10.1186/s13059-015-0832-9) contains supplementary material, which is available to authorized users.

## Background

Mechanisms of transcriptional response mediated by signal-dependent transcription factors (inducible TFs) are not well understood at the level of chromatin topology. Recent genome-wide studies have revealed that the majority of TF binding sites (up to 90 %) are distal to promoters and located in intragenic and intergenic regions [1–9]. These studies collectively revealed cell-type-specific constellations of distal regulatory regions that change during differentiation and development in a highly ordered fashion, whereby some distal regulatory regions are being set up de novo and others are decommissioned. This implies that at least some lineage-specific and/or signal-dependent TFs effectively open the chromatin structure and prepare the chromatin for subsequent binding of other TFs. A simplistic model of how such plasticity can be achieved is that long-range interactions among and between enhancers and promoters are dynamically established or disrupted. Many recent studies have purported an active or “instructive” role of inducible TFs in mediating long-range chromatin contacts for efficient regulation of target genes [10–14]. The orchestrated long-range interaction changes have also been reported in embryonic stem cells (ESC) and ESC-derived lineages [15] on a topological domain (TAD) level. In contrast, other studies suggest a static or “permissive” model in which inducible TFs passively join pre-existing interaction networks of regulatory elements without affecting the organization of long-range interactions [14, 16, 17]. The *HoxD* locus serves as an example of pre-formed long-range interactions [18]. Interestingly, in another report focusing on the *HoxD* locus, the authors directly compared the interaction profiles obtained by chromosome conformation capture (3C)-based methods and fluorescent in situ hybridization. The authors conclude that interactions identified by 3C-based methods at such high resolution do not always represent true proximal ligations, but may be a consequence of indirect cross-linking [19]. Discrepancies between studies on inducible TF-mediated long-range chromatin contacts may be due to differences in resolution and methodology or to the use of asynchronous cells.

Glucocorticoid receptor (GR) is a ligand inducible TF that belongs to the nuclear receptor superfamily [20]. Hormone binding dissociates the GR-containing cytoplasmic complex; GR then translocates to the nucleus where it binds to chromatin to regulate target gene activity. Nuclear factor kappa-b (NFκB) is a heterodimeric TF that regulates various biological processes such as cell growth, development, and the inflammatory response. In response to inflammatory stimuli such as the pro-inflammatory cytokine tumor necrosis factor alpha (TNFα), NFκB dissociates from an inhibitory cytoplasmic complex, translocates to the nucleus, and subsequently regulates its target genes [21–25]. Co-activated GR and NFκB share a large proportion of genomic regulatory elements and co-regulate many genes in a mutual antagonistic or synergistic manner [7, 26–29]. The majority of GR and p65 (a major NFκB subunit) binding events occur at genomic loci that exhibit pre-existing enhancer signatures. In this scenario, TFs other than GR and NFκB have established and maintain an open chromatin conformation, facilitating binding or recruitment of GR and p65 to their binding sites [30–32]. At a minority of GR and p65 binding sites (~10 %), the activated TFs establish de novo enhancer-like loci [5, 33, 34].

To gain insight in how GR and NFκB regulate their target gene repertoire from distal binding sites (DBSs), we mapped the chromatin interactions before and after GR and NFκB activation by generating high-resolution chromatin interaction profiles using the chromatin interaction analysis by paired-end tag sequencing (ChIA-PET) method [35, 36]. We used antibodies against enhancer-associated P300 and against RNA polymerase II (POLII). P300 is a co-factor shared by GR and NFκB and its genomic occupancy in general is considered a hallmark of active enhancers [37–40]. We scrutinized the local chromatin interaction networks at genomic loci that are de novo established and compared them to those of pre-existing loci. We extended our analysis using high-resolution circular chromosome conformation capture (4C) technology on a subset of genomic viewpoints harboring de novo programmed regulatory elements. Collectively, our comprehensive analyses reveal a role of signal-dependent TF-induced dynamic changes in chromatin regulatory networks and its impact on gene regulation.

## Results

### P300 is recruited to latent distal binding sites by ligand activated GR and/or NFκB

**Fig. 1:**
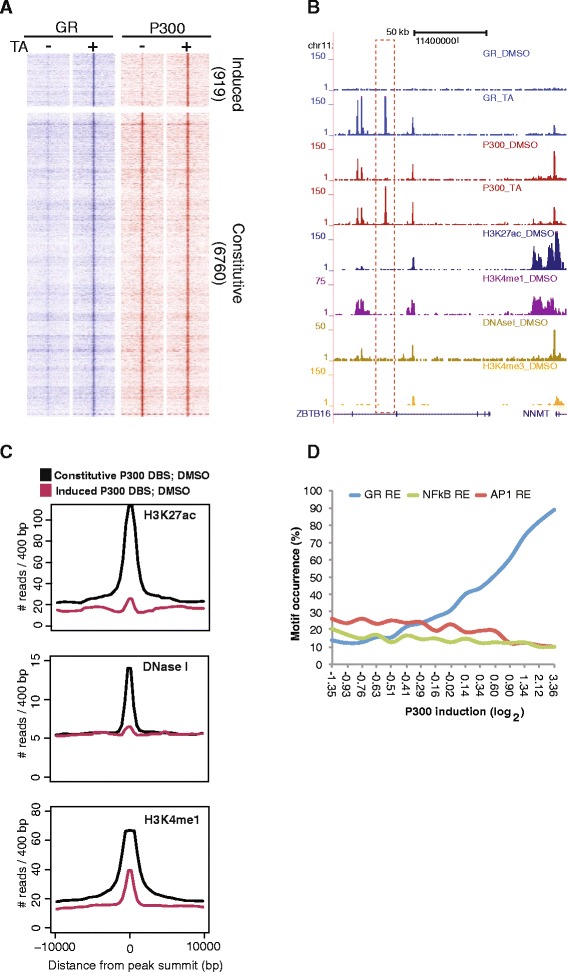
Activated glucocorticoid receptor (*GR*) recruits P300 to epigenomically latent genomic regions. **a** Color profile depicting the GR and P300 signal at all GR-bound regions with either constitutive or ligand (triamcinolone acetonide [*TA*]) -induced P300 occupancy. **b** Example screenshot depicting the TA-induced P300 distal binding site (DBS; *dotted box*) and constitutive P300 DBS. **c** Basal (untreated cells) H3K27ac, DNase I hypersensitive site, and H3K4me1 signal at all GR-induced and constitutive P300 DBSs. **d** Motif occurrence at all GR-bound DBSs presented as a function of TA-dependent P300 recruitment (x-axis). *DMSO* dimethyl sulfoxide, *RE* response elements

To gain insight into the impact of GR activation on the chromatin state and three-dimensional (3D) organization, we first performed chromatin immunoprecipitation followed by deep sequencing (ChIP-seq) for GR, P300, epigenetic marks (H3K27ac, H3K4me3, and H3K4me1) and DNase I accessibility analysis. Ligand-activated GR binds to several thousand genomic loci [5, 7, 8, 41], of which more than 90 % (7679/8303) were located distally (>5 kb) from transcription start sites in HeLa cells (Additional file 1: Figure S1A). The vast majority (6760/7679) of these DBSs were DNase I accessible, bound by P300, and marked with H3K27ac and H3K4me1 prior to hormone stimulation (Fig. 1a–c). We refer to these as “pre-existing” P300 sites. Importantly, a subset of GR DBSs (919/7679) displayed the hallmarks of poised enhancers prior to ligand treatment, being largely inaccessible to DNase I, lowly marked with H3K4me1, and not marked with H3K27ac and P300. Interestingly, P300 was robustly recruited to these epigenetically dormant loci upon GR induction (“induced” P300 sites; Fig. 1a–c). At a smaller subset (529/6760) of GR DBSs, P300 occupancy was moderately reduced upon hormone treatment (data not shown). Next, we analyzed the pre-existing and induced P300 DBSs for TF motifs [7]. As expected, ligand-induced P300 DBS were highly enriched for glucocorticoid response elements (GREs), whereas AP1 was the most prevalent motif detected at pre-existing P300 sites (Fig. 1d). Our observations together with published data [5] suggest that at the induced P300 DBSs that are pre-marked with H3K4me1, GR binds directly to consensus GREs and recruits P300 to set up enhancer-like elements.

P65 was also predominantly bound at distal genomic loci (11,454/12,546) (Additional file 2: Figure S2A), of which the majority (10,453/11,454) were occupied by P300 prior to TNFα stimulation (pre-existing P300 sites). At a subset of p65 DBSs (1001/11,454), P300 was detectable only upon TNFα stimulation (Additional file 2: Figure S2B,C). TNFα-induced P300 DBSs were enriched for the NFκB response element (NFκB-RE) (Additional file 2: Figure S2D). Furthermore, induced P300 DBSs that were barely or not marked by H3K27ac were inaccessible to DNase I, yet displayed readily detectable levels of H3K4me1 prior to TNFα induction and p65 binding (Additional file 2: Figure S2E). In line with a recent study in mouse macrophages [34], we presume that TNFα induction activates poised or latent enhancers. We also observed pre-existing P300 binding at many sites (~25,000) that were not significantly co-occupied by GR or p65 (Additional file 3: Figure S3A,B). These sites likely have a regulatory role in association with other TFs.

Because GR and p65 share a large number of regulatory elements (~30 %) and co-regulate many genes, we performed a similar analysis upon co-activation of GR and p65. We detected all the induced P300 DBSs that were uncovered upon single activation of GR or p65. An additional subset of inducible P300 sites (~700) was unveiled only upon co-stimulation, displaying significantly increased DNase I accessibility and H3K27ac, and a marginal increase in H3K4me1 (Additional file 4: Figure S4, Additional file 5: Figure S5, Additional file 6).

Taken together, GR and p65 mostly join pre-existing enhancer-like P300 DBSs that are set up by other TFs such as AP1. At a subset of latent genomic locations marked with low levels of H3K4me1, GR and/or NFκB binding induces DNA accessibility, recruitment of P300, and H3K27ac deposition. Because the induced P300 sites are highly enriched for their respective consensus response elements, it appears that recruitment of GR and NFκB to their respective cis-acting elements can initiate the formation of an active enhancer configuration, in line with recent studies [5, 33, 34].

### ChIA-PET reveals P300 enhancer interaction networks

Next we focused on long-range chromatin contacts associated with P300 DBSs. We performed chromatin interaction analyses on co-stimulated cells to uncover the largest number of induced P300 DBSs (2881), and contrasted them to vehicle treatment. We performed ChIA-PET, an antibody-based method, to map the genome-wide chromatin interactions at high resolution [35, 36, 42]. We mapped the chromatin interactions using P300 and POLII antibodies. Sequencing of the P300 ChIA-PET libraries yielded 36.7 and 18.2 million uniquely mapped paired-end tags (PETs) for vehicle and co-stimulated samples, respectively. Among these, 1.4 and 1.2 million reads were self-ligation PETs (defined as ligation endpoints or anchors less than 5 kb apart) accounting for 15,148 and 16,366 putative P300 binding sites in vehicle and co-treated libraries, respectively (Additional file 7: Table S1). The vast majority (>90 %) of these self-ligation PETs co-localized with the P300 binding sites identified by ChIP-seq (Additional file 8: Figure S6A). ChIP-seq binding sites with low signal strength were not detected as binding sites in ChIA-PET data sets (Additional file 8: Figure S6B). Therefore, we used ChIP-seq binding sites (identified from ~20 million unique reads) as anchors to identify high confidence chromatin contacts. Ligation PETs that had their anchors between 5 and 1000 kb from each other and co-localized with high confidence P300 ChIP-seq binding sites were defined as long-range interactions. We identified 2363 and 5429 intra-chromosomal interactions using the P300 antibody in vehicle and co-stimulated cells, respectively. Using a similar approach, a large number of intra-chromosomal interactions were detected in a ChIA-PET analysis using a POLII antibody (Additional file 9: Table S2). P300 and POLII ChIP-seq binding sites that were involved in chromatin interactions were of higher signal strength compared to those not detected in chromatin interactions (Additional file 8: Figure S6C).

**Fig. 2:**
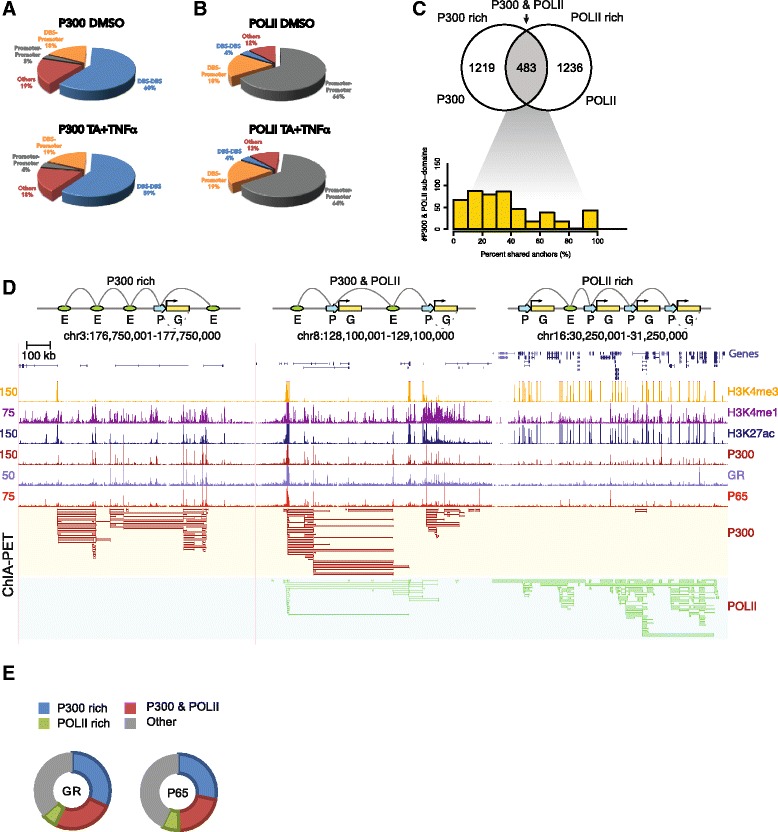
P300 ChIA-PET interaction profile shows an enhancer-centered interaction pattern in contrast to promoter-centered RNA polymerase II (*POLII*) interactome. **a** Proportion of distal binding site (*DBS*)-promoter, promoter-promoter, and DBS-DBS interactions identified by P300 ChIA-PET in cells treated by vehicle (dimethyl sulfoxide [*DMSO*]) (*upper panel*) or triamcinolone acetonide (*TA*) + tumor necrosis factor alpha (*TNFα*) (*lower panel*). **b** Proportion of DBS-promoter, promoter–promoter, and DBS–DBS interactions identified by POLII ChIA-PET in cells treated with vehicle (*upper panel*) or TA + TNFα (*lower panel*). **c** Venn diagram depicting the extent of overlap between P300 interaction subdomains and POLII interaction subdomains (*upper panel*). Histogram depicting the percentage of P300 and POLII shared anchors in P300 & POLII interaction subdomains (*lower panel*). **d** Example screenshots of P300-rich (*left panel*), P300 & POLII-rich (*middle panel*), and POLII-rich (*right panel*) interaction subdomains depicting the ChIP-seq and ChIA-PET interaction data. **e** Distribution of glucocorticoid receptor (*GR*) (*left panel*) and p65 (*right panel*) binding sites in P300 rich, P300 & POLII-rich, and POLII-rich interaction subdomains

The majority of P300-associated long-range interactions occurred between distal regulatory elements (DBSs, ~60 %), whereas about 20 % occurred between promoters and DBSs (Fig. 2a). In contrast to the P300 interactome, POLII-associated interactions were found predominantly between promoters (64 %) and only 19 % involved DBS-promoter interactions (Fig. 2b). Visual inspection suggested that identified chromatin interactions occurred frequently between a multitude of P300 DBSs that aggregate into interaction subdomains (Additional file 8: Figure S6D) similar to replication or TADs [43, 44]. Indeed, more than 95 % of all P300 and POLII long-range interactions were confined to such domains as defined by DNA replication timing in HeLaS3 cells [44, 45] (Additional file 8: Figure S6E). Whereas the average TAD length is ~1.7 Mb, the average widths of P300 and POLII subdomains were 118 kb and 96 kb, respectively. Direct comparison of individual P300 and POLII interaction domains revealed that two fifths (39.6 %) overlapped, whereas the remainder appeared to involve only P300 or POLII (Fig. 2c, upper panel). The degree of P300 and POLII anchor overlap in P300 and POLII shared interaction domains varied, with most of the subdomains sharing less than 50 % of anchors (Fig. 2c, lower panel). Representative examples of P300-rich, P300 and POLII, and POLII-rich interaction subdomains are shown in Fig. 2d.

Because GR and p65 preponderantly bind to putative enhancers that are marked with P300, it would be expected for GR and p65 binding sites to be enriched within the P300 ChIA-PET interaction network. Indeed, about 60 % of GR and 50 % of p65 binding sites were located within P300 centric interaction subdomains (P300 rich, P300 & POLII). POLII-rich promoter–promoter networks were largely devoid of GR and p65 binding events (Fig. 2d,e).

### Ligand treatment enhances long-range interactions at induced P300 distal binding sites

Next we set out to investigate whether pre-existing and induced P300 sites participate equally in long-range chromatin interactions. Upon ligand activation we observed a significant gain of DNaseI accessibility and active chromatin marks at induced P300 DBSs. We reason that these sites might have an increased interaction upon ligand activation.

**Fig. 3:**
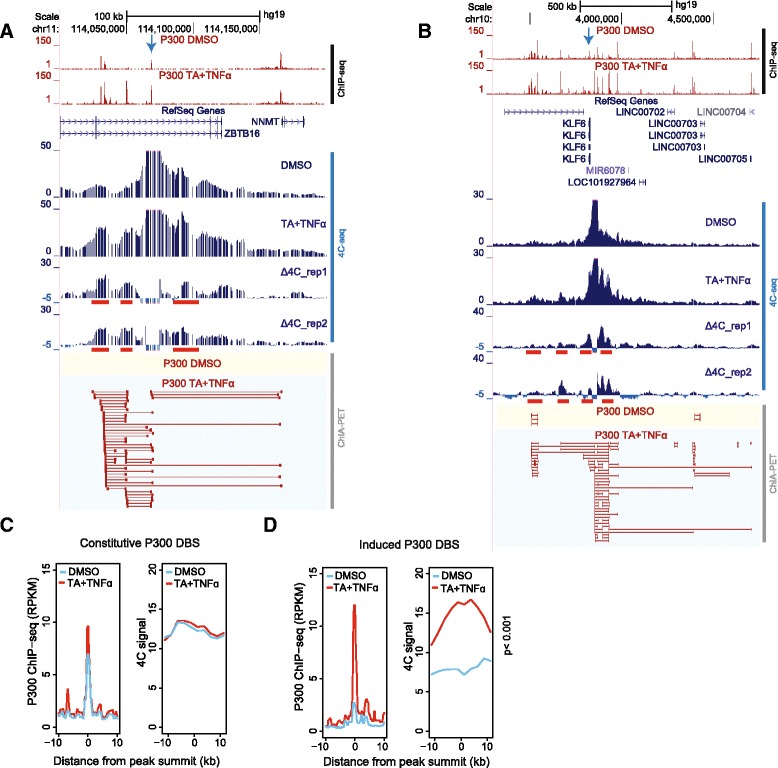
Glucocorticoid receptor (GR)-dependent and p65 activation-dependent changes in chromatin interactions. **a**, **b** Direct comparison of chromatin contacts revealed by P300 ChIA-PET and 4C-seq analyses at the ZBTB16 (a) and KLF6 loci (b). 4C-seq bait loci are marked on each screenshot (*blue arrow*). Genomic regions that show a marked change in 4C signal upon triamcinolone acetonide (*TA*) + tumor necrosis factor alpha (*TNFα*) treatment are marked *red* below the Δ4C track. **c** Direct comparison of changes in average ChIP-seq signal (*left panel*) and 4C signal (*right panel*) at all the constitutive P300 distal binding sites (*DBS*s) within the ten 4C-seq genomic view points upon TA + TNFα treatment. **d** Direct comparison of changes in average ChIP-seq signal (*left panel*) and 4C signal (*right panel*) at all the induced P300 DBSs within the ten 4C-seq genomic view points upon TA + TNFα treatment. *DMSO* dimethyl sulfoxide

To validate the P300-mediated long-range interactions and to gain insight into their frequency, we selected 4C viewpoints in eight different P300 interaction subdomains that encompassed 58 different genomic loci (anchors) in our ChIA-PET analysis. 4C-seq libraries from at least two independent biological replicas per viewpoint were sequenced to obtain more than 2 million high-quality, uniquely aligned reads (Fig. 3, Additional file 10: Figure S7, Additional file 11: Figure S8, Additional file 12: Figure S9). This sequencing depth is regarded adequate to map all ligation events within the viewpoint [46].

In the *ZBTB16/NNMT* locus (Fig. 3a), transcription of the *NNMT* gene was induced by co-stimulation. This locus contained one ligand-induced P300 binding site that also gained H3K27ac and DNase I accessibility (see also Fig. 1b) and three pre-existing P300 sites. Using one of the pre-existing P300 sites as the viewpoint in 4C experiments, we detected its interaction with other pre-existing P300 sites. Upon ligand activation, we observed the formation of novel interactions involving the ligand-induced P300 DBS as well as a general increase in the interaction signal at pre-existing sites. In ChIA-PET, we detected interactions between all the enhancers only upon ligand induction.

The *KLF6* locus encompassed multiple constitutive and four induced P300 binding sites (Fig. 3b). Upon stimulation, transcription of the *KLF6* gene was highly induced and multiple enhancers gained P300, H3K27ac, and DNase I accessibility. A GR-induced DBS was used as the viewpoint for 4C. In vehicle-treated cells, we detected weak 4C signals between the bait and surrounding pre-existing and induced P300 DBSs. These contacts were robustly increased upon co-stimulation. An additional six genomic viewpoints showed a similar increase in interaction frequencies and inclusion of induced P300 binding sites in the interaction network upon ligand induction (Additional file 11: Figure S8, Additional file 12: Figure S9).

To assess the interaction frequency at P300 DBSs, we divided the P300 DBSs that were detected in our 4C analysis (eight viewpoints) into induced and pre-existing. For each group we plotted the average of P300 ChIP-seq and 4C signal (reads per kilobase per million mapped reads [RPKM]) in control and stimulated cells. The constitutive P300 binding sites displayed a similar ChIP-seq and 4C signal pattern in vehicle-treated and ligand-treated cells (Fig. 3c). Importantly, induced P300 binding sites showed a significantly higher (*p* < 0.001, t-test) 4C signal in ligand-treated cells than the control cells (Fig. 3d).

### GR and NFκB activation enhances long-range chromatin contacts

The 4C assays support the presence of long-range interaction networks among P300 DBSs. Furthermore, they uncovered a significant increase in contact frequency at induced but not at pre-existing P300 DBSs (Fig. 3c,d). To further investigate this difference, we divided the ChIA-PET interaction subdomains into two groups: subdomains containing only pre-existing P300 DBSs, and subdomains containing at least one induced P300 DBS. We then compared their interactome in the ChIA-PET profiles. However, to directly compare the two conditions, the immunoprecipitation-introduced bias inherent to ChIA-PET had to be taken into account. The ChIP step results in a restricted representation of the interactome. A possible confounding factor in ChIA-PET is that chromatin regions with a higher number of binding sites with high occupancy (RPKM) – that is higher local concentration of P300 – may be ChIPed with higher efficiency than regions with fewer binding sites and lower P300 occupancy.

**Fig. 4:**
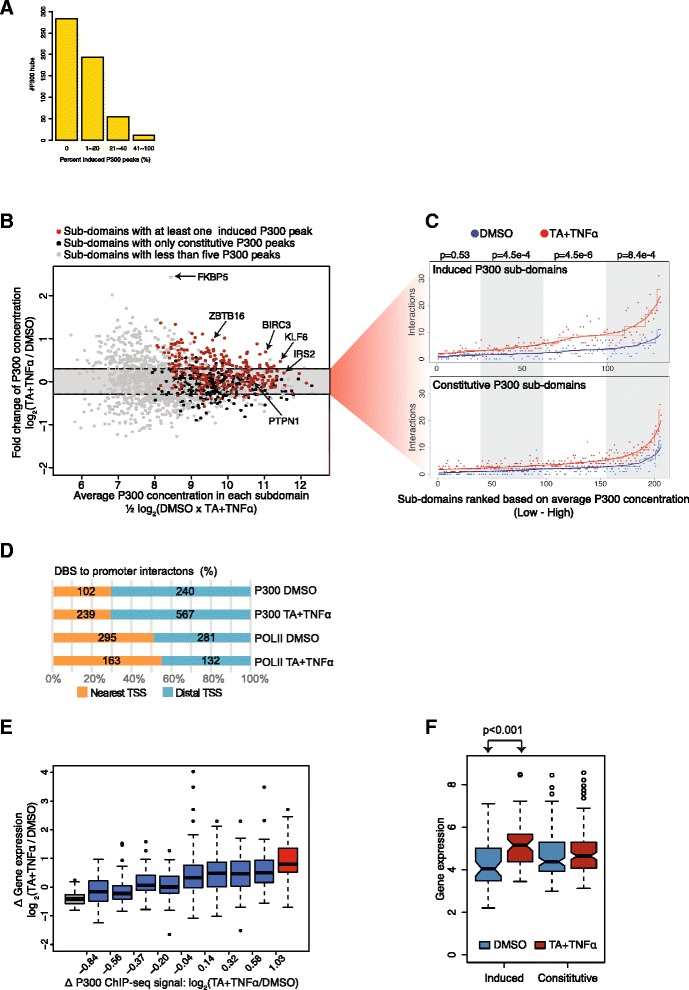
Ligand-induced enhancement of chromatin contacts within P300 interaction subdomains. **a** Bar plot depicting the percentage of induced P300 peaks in the P300 hubs with at least five P300 peaks. **b** P300 local concentration at all P300 interaction subdomains that are ordered based on average P300 density (x-axis) and ligand-induced change in P300 concentration (y-axis). Sub-domains harboring at least five P300 distal binding sites (*DBS*) are presented**. c** Total number of chromatin interactions detected at each interaction subdomain upon vehicle dimethyl sulfoxide (*DMSO*, *blue dots*) and triamcinolone acetonide (*TA*) + tumor necrosis factor alpha (*TNFα*, *red dots*) treatment is presented. Sub-domains that show no more than 25 % change in total P300 concentration (reads per kilobase per million mapped reads) that either harbor at least one induced P300 DBS (*upper panel*) and only constitutive P300 DBS (*lower panel*) are used. *p*-values (Mann–Whitney) were generated by comparing interaction ratios (TA + TNFα/DMSO) between the induced and constitutive subdomains that are within each bin (shaded area). **d** Proportion of DBSs to nearest first gene promoters or DBSs to distal gene promoter interactions identified by P300 and RNA polymerase II (*POLII*) ChIA-PET analysis. *TSS* transcription start site. **e** Co-activation-dependent changes in expression of genes that are directly looped to P300 DBSs. P300 DBSs were initially ordered according to agonist-induced P300 recruitment (low to high) and subsequently divided into 10 equal bins. The average transcriptional change of genes in each bin is presented. Expression of genes that are in bins harboring significantly repressed (*gray*), induced (*red*), and constitutive (*blue*) P300 DBSs upon co-stimulation. **f** Co-activation-induced changes in expression of genes that are within interaction subdomains harboring either induced or only constitutive P300 DBSs

In order to accurately compare the pre-existing and induced subdomains in untreated and co-stimulated ChIA-PET libraries, we first estimated the local P300 concentrations (average P300 signal) by summing up the RPKM values of P300 DBSs in ChIA-PET interaction subdomains harboring at least five P300 DBSs with a different degree of P300 induction (Fig. 4a). With few exceptions, the co-stimulation marginally affected the local concentration of P300 as compared to vehicle-treated cells (<2-fold) (Fig. 4b). Next, we selected subdomains that upon co-stimulation responded with no more than a 25 % change in total P300 concentration (subdomains within the shaded area in Fig. 4b). We computed the chromatin interaction frequencies (ChIA-PET interactions/subdomain) in subdomains that had at least one or no induced P300 DBSs. In order to take into account the coverage difference of the two P300 ChIA-PET libraries, we used the one-sided Mann–Whitney–Wilcoxon test. Interestingly, P300 hubs bearing induced P300 DBSs displayed a significant increase in chromatin contacts upon co-stimulation versus vehicle-treated cells (Fig. 4c, upper panel). Such preference is not evident in subdomains harboring only pre-existing P300 DBSs (Fig. 4c, lower panel).

Thus, preferential involvement of ligand-induced P300 DBSs in chromatin interactions implies that GR and/or NFκB binding to DNA via their cognate cis-acting elements opens up the closed chromatin by recruiting chromatin-remodeling complexes. Such open chromatin regions preferentially contact other P300 DBSs with a similar regulatory factor composition and chromatin state, resulting in an interaction network that is synchronized upon ligand-dependent GR and/or NFκB recruitment, resulting in increased contact frequency. We preferentially detected the networks that were highly affected by ligand stimulation. These networks are already established prior to stimulation (as detected by 4C-seq). However, the increase of the contact frequency at induced P300 DBSs upon ligand activation indicates the synchronization of such networks.

We therefore consider that the (over) representation of induced interactions in our data sets may imply that a considerably larger proportion of cells in the population have the P300 protein network at these loci because the queried loci were synchronized by ligand treatment.

### GR and NFκB networks are enriched with their target genes

Finally, we assessed the impact of agonist-induced regulatory elements and their special re-organization on transcriptional regulation using gene-body POLII density as a direct read-out [6]. We quantified all the DBS-promoter contacts that were identified in both P300 and POLII interaction data sets. Importantly, 70 % of P300 and 50 % of POLII bound DBS were not contacting the nearest (first) active transcription start site (TSS) but a more distal TSS (Fig. 4d). Next, we computed the gene-body POLII density of genes that were connected to (induced and pre-existing) P300 DBSs. We observed a consistent positive correlation between ligand-dependent gene induction and P300 induction at the DBS (Fig. 4e). Genes linked to induced P300 DBSs respond avidly to ligand induction as compared to genes that are interacting with pre-existing P300 DBSs. Similarly, expression of all genes in the agonist-induced interaction subdomains was significantly increased upon co-stimulation whereas genes in the constitutive subdomains were unresponsive to agonists (Fig. 4f). Gene ontology (GO) analysis of genes in the induced P300 hubs instigated by activation of NFκB (TNFα or co-stimulated) are enriched for GO terms associated with inflammatory response whereas genes linked to activated GR-induced P300 hubs are enriched for various biological processes, including macromolecule metabolic processes. Genes connected to constitutive P300 hubs are enriched for GO terms associated with general cellular processes (Additional file 13: Table S3). Taken together, our data strongly suggests that GRE-containing or NFκB RE-containing latent enhancers that are activated upon ligand stimulation preferentially engage the GR and NFκB target genes and subsequently modulate their expression.

## Discussion

In this study, we have analyzed the impact of agonist-activated GR and p65 (NFκB) on chromatin state, 3D organization, and transcriptional regulation. Activated GR and p65 are mainly recruited to pre-existing regulatory elements that are pre-bound by P300, and display the epigenetic signature of active enhancers prior to TF activation, that is, they fall into the “permissive” model category. Our findings are in line with recent findings suggesting that signal-dependent TFs largely access the enhancer landscape that is set up by other lineage-specific TFs such as PU.1, C/EBPα, and AP1 [4, 5, 30, 34]. However, in ChIP-seq, we also observed de novo recruitment of P300 by activated GR and/or p65 to thousands of regions that subsequently acquired enhancer-like epigenetic features, in line with recent studies [5, 33, 34]. ChIA-PET and 4C analysis indicate that these sites are involved in interaction that would fall into the “instructive” category. The chromatin signature and epigenetic changes in response to ligand activation at these induced DBSs are reminiscent of “latent enhancers” [34, 47].

An important question is how the agonist-induced enhancers and their target genes are spatially organized. We have compared the P300-mediated chromatin interactomes before and after GR and NFκB co-activation. Using ChIA-PET against P300, we observed the formation of P300-mediated long-range interactions at subdomains bearing induced P300 DBSs in response to ligand activation. With the current depth of ChIA-PET libraries, these interactions appeared to be either formed de novo (from latent enhancers) or stabilized upon ligand induction. In 4C analysis we reproducibly detected increased interactions at induced P300 DBSs; the induction in the 4C approach is, however, less pronounced as compared to ChIA-PET. This difference is likely due to intrinsic differences between the two assays: 4C detects long-range interactions irrespective of the presence or absence of P300 and hence is able to detect lower strength or lower frequency interactions and those that are P300 independent. In ChIA-PET, the immunoprecipitation step enriches for interactions mediated by P300 and does not pick up P300 independent interactions, providing an all or nothing picture. Collectively and in agreement with each other, our ChIA-PET and 4C results show that activation of GR and/or NFκB facilitates an induced interaction signal at a subset of DBSs. We interpret this increased signal as an increase in either the interaction frequency (stabilization of a network) or in the proportion of cells that engage in such interaction (synchronization of a network).

Recent 3C-based studies of individual loci reported on the role of GR and NFκB in long-range gene regulation [10, 13, 48]. For example, the *Lcn2* gene locus is engaged in multiple long-range contacts with GR DBS. In agreement with our findings, it was shown that activated GR increases local chromatin interactions without dramatic change in 3D organization. In another report exploiting the 4C approach, activated GR was shown to bind a downstream enhancer of the *Tsc22d3* gene, causing a 2-fold increase in long-range enhancer–promoter interaction and activation of transcription [13]. Similarly, TNFα induces chromatin interactions between distal NFκB-bound enhancers and the promoter proximal regulatory sites of *CCL2* [10]. In contrast, a recent study based on genome wide Hi-C analysis revealed that the vast majority of TNFα responsive enhancers, as determined by p65 binding, show little change in DNA looping after TNFα treatment [16]. The authors note that only ~15 % of p65 DBSs display an activated enhancer signature (increase in H3K27ac signal and enhancer RNA production) upon TNFα treatment. The apparent discrepancy with our study is likely due to the differences in resolution of applied techniques. In agreement with Jin et al., we found that the majority of long-range interactions are pre-established and not dynamic; however, by applying ChIA-PET and high-resolution 4C, we found a significant increase in long-range interactions at induced but not constitutive P300 DBSs. These changes in a subset of interactions are conceivably difficult to pick up using a relatively low-resolution Hi-C-only approach.

One of the questions debated in the field of chromatin topology is the extent to which long-range interactions are dynamic and correlate with gene expression, such as in response to extracellular stimuli or during differentiation. The instructive model suggests de novo formation of long-range interactions, where lineage-specific and/or signal-induced TFs establish a new interaction landscape and affect the expression of their target genes [10–14, 49]. Our data provides support for this model: at ligand-induced DBSs, ChIA-PET and 4C data show an increased interaction signal at loci that were largely closed with low or no active epigenetic marking (H3K27ac) but with low levels of H3K4me1, reminiscent of latent enhancers [34]. Our data also provide support for the permissive model, showing that the long-range interaction landscape is pre-formed in the absence of ligand induction. Ligand-activated TFs appear to join a pre-set network of enhancers and trigger transcription by lineage-specific and/or signal-induced TFs [16–18].

## Conclusions

We conclude that the ligand-activated GR and p65 induce chromatin accessibility, P300 recruitment, and alterations of 3D chromatin structure at a subset of genomic loci. At subdomains with induced P300 binding, activated GR and p65 facilitate close spatial proximity of the induced P300 DBS with a pre-existing interaction network and enhancement of 3D chromatin contacts. Our data suggest that ligand induction causes synchronization or stabilization of active chromatin states and higher order structure in a large proportion of cells to facilitate efficient regulation of their target genes. We speculate that this spatial clustering of regulatory elements can cause an increase in the local concentration of regulatory proteins, which ultimately can enhance the transcriptional activity of associated genes. Further experiments are needed to validate and expand on these findings, to elucidate the role of inducible TFs in long-range regulation, and to firmly establish that increased physical looping interaction indeed leads to increased transcription.

## Methods

### Cell culture

HeLa B2 cells were maintained as described [7]. Cells were cultured in Dulbecco’s Modified Eagle Medium supplemented with 10 % charcoal stripped fetal calf serum for 72–96 h before subsequent treatment and/or harvesting. Cells were treated with either DMSO or 1 μM of TA (T6501, Sigma-Aldrich, St. Louis, Missouri, United States) for 4 h with or without an additional treatment with 10 ng/mL TNFα (T0157, Sigma-Aldrich) for the last hour.

### ChIP-seq

ChIP was performed according to standard protocol [50] with minor modifications. Paraformaldehyde (1 %) cross-linking was carried out for 10 min followed by the chromatin preparation as described earlier [7]. Nuclei were re-suspended in ChIP-incubation buffer at a concentration of 20 × 10^6^ cells/mL and sheared (seven cycles with each cycle containing 10 s power on and 10 s interval) using Bioruptor®Plus (B01020001, Diagenode, Liege, Belgium). Sonicated chromatin equivalent of 4 × 10^6^ cells was incubated with relevant antibody overnight at 4 °C. Antibodies against P300 (sc-585x, Santa Cruz Biotechnology, Inc., Dallas, Texas, United States), POLII (MMS-126R-500, Covance, Inc., Princeton, New Jersey, United States), H3K27ac (C15410196, Diagenode), H3K4me1 (C15410194, Diagenode), and H3K4me3 (C15410003, Diagenode) were used. ChIP-seq sample preparation and sequencing was performed according to manufacturer’s instructions (Illumina, San Diego, California, United States) and essentially as described [6, 9, 51] (http://www.blueprint-epigenome.eu).

### ChIP-seq data analysis

The image files generated by HiSeq2000 (Illumina) were processed to extract sequence data and the 36/42 bp tags were unambiguously mapped to the human genome (NCBI, hg19) using the bwa aligner, allowing at most one nucleotide mismatch. Reads were further directionally extended to 200 bp, corresponding to the original length of the DNA fragments used for sequencing. For each base pair in the genome, the number of overlapping sequence reads was determined, averaged over a 10 bp window, and visualized in the University of California Santa Cruz genome browser (http://genome-euro.ucsc.edu). ChIP-seq data sets were normalized as described [6, 7] in order to eliminate the differences caused by sequencing depth/mapping efficiency.

Detection of putative P300 and POLII binding sites was performed using MACS (version 1.4.2) [52] with the *p*-value <10^−9^. Peaks identified by using each antibody in DMSO, TA, TNFα, and TA + TNFα were combined in a common pool and sequence tags were counted under each peak location (for each data set separately). Then we calculated the intensity (log_2_ RPKM) of peaks in each treatment. Binding sites that showed a significant change (median ± 2 × median absolute deviation; *p* < 0.05) in signal for P300 or POLII in a treatment compared to that in the vehicle-treated sample were regarded as dynamic binding sites. Published GR, p65, and POLII ChIP-seq data that were generated in an identical experimental setup in HeLa B2 cells [GEO: GSE24518] were used in this study.

### DNase I-seq

DNase I libraries were prepared from DMSO-treated and TA + TNFα-treated Hela B2 cells as described (http://www.uwencode.org/protocols). In brief, 5 × 10^6^ nuclei were isolated using Buffer A (15 mM NaCl; 60 mM KCl; 1 mM EDTA, pH 8.0; 0.5 mM EGTA, pH 8.0; 15 mM Tris–HCl, pH 8.0; 0.5 mM spermidine) supplemented with 0.06 % IGEPAL CA-630 detergent. DNase I treatment (60 units) was performed for 3 min and the reaction stopped with stop buffer (50 mM Tris–HCl, pH 8; 100 mM NaCl; 0.10 % SDS; 100 mM EDTA, pH 8.0; 1 mM spermidine; 0.3 mM spermine). The sample was further fractionated on 9 % sucrose gradient for 24 h at 25,000 rpm at 16 °C. Fractions containing DNA fragments smaller than 1 kb were purified and processed for sequencing according to the Illumina library preparation protocol. Normalized (read number equalized) DNase I data sets were used for the downstream analysis and visualization.

### ChIA-PET library preparation

ChIA-PET libraries were prepared using the standard protocol [35, 36]. Chromatin preparation and ChIP enrichment using P300 and POLII antibodies were performed as described above. Briefly, chromatin captured on magnetic beads was trimmed (blunt end), phosphorylated on 5′ ends, then underwent biotinylated half-linker ligation. Chromatin complexes were then divided into two equal halves and two independent half-linker ligation reactions were performed using half-linkers A and B containing specific barcodes (linker-A TAAG; linker-B ATGT). Subsequently, chromatin complexes were eluted from the beads and two linker ligation aliquots were combined together for proximity ligation under diluted conditions. Subsequently, reverse cross-linked and purified circular DNA was digested using MmeI enzyme (the restriction site is encoded on the linker). Next, biotinylated DNA fragments were immobilized on M-280 streptavidin Dynabeads (Invitrogen, Carlsbad, California, United States) followed by adaptor ligation. The efficiency of the library preparation was evaluated by polymerase chain reaction (PCR) and subsequent gel electrophoresis. Next, each library characterized by adaptor-ligated DNA fragments carrying 20 bp of genomic DNA flanking the 36-bp linker sequence on either side was sequenced on a HISeq200 (Illumina). A typical sequencing run yielding 200 million single-end reads of 100-bp length was generated for each library.

### ChIA-PET data analysis

The first 72 bp of each sequenced read carrying the complete ChIA-PET ligation product (linker plus genomic DNA) was taken for the further analysis after trimming the ends of each read. Subsequently, single-end sequenced reads were split at the linker ligation junction (linkerA/B-|-linkerA/B) and flipped to make the data compatible (similar to paired-end sequencing reads) for the ChIA-PET data analysis pipeline [42]. The average distance between binding sites (P300 and POLII), identified based on the ChIA-PET self-ligation PETs and the binding sites identified by ChIP-seq, were examined to ascertain the reproducibility of binding sites by these methods. The binding sites identified by both methods were highly comparable, but a larger number of total binding sites were identified by ChIP-seq owing to the higher sequencing depth. Therefore, we used ChIP-seq binding sites as anchors to identify the intra-chromosomal and inter-chromosomal interaction PETs. True long-range interaction signals were distinguished from the non-specific technical interaction noise by using the method described earlier [42]. Briefly, interaction PETs having a PET count equal to two or more for P300 libraries and three or more for POLII libraries at a false discovery rate <0.05 were considered as high confidence interaction clusters. We used a 5 kb and 1 Mb genomic span as the lower and upper cutoff limits, respectively, to define the high confidence interaction PET data. Each interaction PET contained a pair of interacting anchors. Direct overlap (book end or 1 bp) of anchors of each cluster with that of other clusters was performed to identify interaction complexes or interaction subdomains. Hence, the interaction clusters were further collapsed in to interaction complexes/subdomains based on the interconnectivity of the PET clusters.

### Identification of dynamic interactions using ChIA-PET data sets

To minimize the bias induced by local P300 concentration on the chromatin interactions detected by ChIA-PET, we analyzed the changes of interaction frequencies per each P300 ChIA-PET defined subdomain as follows. For each subdomain, we counted the number of P300 peaks and calculated the P300 concentration (average log_2_ RPKM) in DMSO-treated and TA + TNFα-treated samples. All the subdomains are ranked by the average P300 concentration of DMSO and TA + TNFα treatments. We discarded the subdomains with less than five P300 peaks, and separated subdomains with at least one induced P300 peak (261 subdomains) and the ones with only constitutive P300 peaks (283 subdomains). We plotted the number of interaction clusters identified in DMSO and TA + TNFα data sets separately for individual subdomains. The subdomains were further filtered by the fold change of P300 concentration (>−0.3 and <0.3) and this resulted in 131 and 206 subdomains, respectively. The Mann–Whitney test was adopted to investigate the agonist-induced change in average chromatin interaction frequencies in comparable groups of subdomains that harbor only constitutive P300 DBSs against those having at least one agonist-induced P300 DBS.

### 4C-seq library preparations

4C assays were performed as described previously [46] with minor modifications. Briefly, 10^7^ cells were cross-linked for 10 min with 2 % paraformaldehyde, quenched with glycine, and lysed in 50 mL lysis buffer (50 mM Tris, pH 7.5; 150 mM NaCl; 5 mM EDTA; 0.5 % NP-40; 1 % TX-100; 1X protease inhibitors) for 30 min. Nuclei were then digested by DpnII enzyme followed by inactivation of the restriction enzyme by incubating at 65 °C for 20 min. The digested chromatin was subsequently ligated (circularized) overnight at 16 °C with 50 U T4 ligase. Ligated chromatin was then reverse cross-linked by incubating with proteinase K at 65 °C and the RNA was removed by additional incubation at 37 °C with RNase A. The purified DNA was further digested with a second restriction enzyme of choice (BfaI, MseI, or NlaIII) followed by circularization of the DNA. The 4C product was subsequently amplified with bait-specific inverse primers (Additional file 14: Table S4). From each 4C library, about 3200 or 800 ng DNA was amplified in multiple parallel PCR reactions containing 200 ng of DNA each, which were subsequently pooled and purified. Amplified bait-containing DNA fragments were ligated to NextFlex DNA barcoded adaptors (Bioo Scientific, Austin, Texas, United States). Adaptor-ligated DNA was purified by Agencourt AMPure XP purification system (Beckman Coulter, Brea, California, United States), PCR amplified (eight cycles), and sequenced single-end on the Illumina HiSeq2000 to obtain 50-bp-long reads.

### 4C-seq data analysis

To improve the mappability of the sequence reads, we generated a reduced genome by extracting the sequences flanking the DpnII sites (30 bp on each strand from the DpnII sites to downstream) based on build version hg19 of the human genome. Then we estimated the mappability of the extracted sequences (each strand separately) and only uniquely mappable DpnII sites were considered for downstream analysis.

All the reads from each library were parsed based on the bait-specific primer sequence and mapped to the reduced genome using bwa (version 0.6.2) with the default parameters. The mapping data of the individual libraries are summarized (Additional file 15: Table S5). We initially mapped each replicate library separately and merged the replicate libraries based on their quality. The 4C signal was calculated using a sliding window of 10 kb (±5 kb of a given DpnII site) and normalized to the total number uniquely mapped reads. Δ4C is the difference of 4C signal in each genomic bin (10 kb) between the normalized DMSO and TA + TNFα data sets.

### Gene ontology analysis

GO analysis was performed using the DAVID web tool [53, 54]. Gene sets were analyzed for enriched GO terms (biological processes) compared to the human genome database as background. Fisher’s exact test was used to identify significantly enriched GO terms.

### Data availability

All the ChIP-seq, ChIA-PET, and 4C raw data files have been submitted to GEO database [GEO: GSE61911]. Previously published GR, p65, and POLII ChIP-seq data can be accessed via [GEO: GSE24518].

### Ethical approval

No approvals were required for the study, which complied with all relevant regulations.

## Additional files

Additional file 1: Figure S1. GR binds predominantly to distal binding sites. Pile-up heat map depicting the H3K4me1 and H3K4me3 signal around (±12 kb) all GR-bound promoters and DBSs. (PDF 272 kb) Additional file 2: Figure S2. Activated p65 induces de novo P300 depositions to latent genomic loci. **(A)** Pile-up heat map depicting the H3K4me1 and H3K4me3 signal around (±12 kb) all p65-bound promoters and DBSs. **(B)** Pile-up heat map depicting the p65 and P300 signal at all p65-bound enhancers upon vehicle, DMSO **(−),** and TNFα (**+**) treatment. **(C)** Example screenshot depicting TNFα-induced P300 recruitment at genomic regions (red box) and recruitment of p65 at genomic loci that are pre-marked by P300. **(D)** Motif occurrence at all p65-bound DBS presented as a function of TNFα-dependent P300 recruitment (x-axis) (top-panel). Level of shared binding of p65 and other TFs at all p65-bound DBS, presented as a function of TNFα-dependent P300 recruitment (bottom panel). **(E)** Level of H3K27ac, DNase I hypersensitivity, and H3K4me1 at all p65-bound DBSs (induced and constitutive P300 sites). (PDF 909 kb) Additional file 3: Figure S3. Large numbers of P300-bound loci are not co-occupied by GR or p65. **(A)** Average ChIP-seq signal of GR and P300 around (±10 kb) all the P300 binding sites that do not show a significant GR occupancy. **(B)** Average ChIP-seq signal of p65 and P300 around (±10 kb) all the P300 binding sites that do not show a significant p65 occupancy. (PDF 168 kb) Additional file 4: Figure S4. Complex epigenetic changes induced by co-activated GR and p65. An example screenshot depicting the GR-dependent (blue box), p65-dependent (red box), and co-stimulation-dependent (black box) induced P300 DBSs and constitutive DBSs. Dynamic changes in DNase I accessibility, epigenetic modifications, and RNA-POLII activity on genes upon co-activation are noticeable characteristics of this locus. (PDF 236 kb) Additional file 5: Figure S5. Co-activation of GR and p65 induces additional de novo P300 DBSs. **(A)** Pile-up heat map depicting signal of P300, GR, and p65 at three groups (p65 dependent, GR dependent, and co-stimulation dependent) of inducible P300 DBSs that were identified upon co-activation of GR and p65. Motif occurrence (%) at all three clusters of induced P300 DBSs (bar graphs). **(B)** H3K27ac, DNase I accessibility, and H3K4me1 signal at all induced P300 DBSs upon vehicle (DMSO) and co-treatments (TA + TNFα). **(C)** H3K27ac, DNase I accessibility, and H3K4me1 signal at all constitutive P300 DBSs upon vehicle (DMSO) and co-treatments (TA + TNFα). (PDF 1137 kb) Additional file 6:
**Supplementary text and Supplementary references.** Cross-talk between GR and NFκB leads to complex changes in chromatin landscape. (DOCX 73 kb) Additional file 7: Table S1. Overview of sequenced and mapped reads of all the ChIA-PET libraries. (XLS 28 kb) Additional file 8: Figure S6.
**(A)** Histogram depicting the genomic proximity (localization) of P300 binding sites identified by ChIP-seq in relation to those identified by using ChIA-PET self-ligation PETs. Identical comparison is performed for both DMSO-treated and TA + TNFα-treated data sets**. (B)** P300 ChIP-seq signal at P300 binding sites commonly identified by ChIP-seq and ChIA-PET and those binding sites that were uniquely detected in the ChIP-seq data set. **(C)** P300 ChIP-seq signal at P300 binding sites that were either involved (anchor) or not involved (non-anchor) in long-range interaction as identified by ChIA-PET analysis. **(D)** An example screenshot depicting the P300 interaction subdomains, P300 ChIP-seq binding sites in relation to topological domains as defined by replication timing data (www.replicationdomain.org). **(E)** Localization of all the interaction subdomains identified by ChIA-PET analysis (P300 and POLII) in relation to topological domains. (PDF 1041 kb) Additional file 9: Table S2. List of all significant long-range interactions identified by P300 and POLII ChIA-PET analysis. (XLS 2165 kb) Additional file 10: Figure S7. Reproducibility of 4C-seq biological replicates at the *FKBP5* locus. (PDF 1094 kb) Additional file 11: Figure S8. Direct comparison of long-range interactions identified by P300 ChIA-PET and 4C-seq analyses at *FKBP5*, *DUSP1*, and *BIRC3* loci. (PDF 1037 kb) Additional file 12: Figure S9. Direct comparison of long-range interactions identified by P300 ChIA-PET and 4C-seq analyses at *PTPN1*, *SULF1* and *ZFHX3* loci. (PDF 1147 kb) Additional file 13: Table S3. Overview of enriched GO terms for the genes that are in various types of interaction subdomains. (XLS 23 kb) Additional file 14: Table S4. Overview of restriction enzyme combinations and inverse PCR primer pairs used for 4C-seq. (XLS 31 kb) Additional file 15: Table S5. Overview of sequenced and mapped reads of 4C-seq libraries. (XLS 31 kb)

## Abbreviations

bp: base pair
ChIP: chromatin immunoprecipitation
DBS: distal binding site
ESC: embryonic stem cells
GO: gene ontology
GR: glucocorticoid receptor
GRE: glucocorticoid response element
kb: kilobase
Mb: megabase
NFκB: nuclear factor kappa-b
PCR: polymerase chain reaction
PET: paired-end tag
POLII: RNA polymerase II
RPKM: reads per kilobase per million mapped reads
TA: triamcinolone acetonide
TAD: topologically associated domain
TF: transcription factor
TNFα: tumor necrosis factor alpha
TSS: transcription start site
