# Supplementary figures and images for: Glucocorticoid receptor and nuclear factor kappa-b affect three-dimensional chromatin organization
Source: Genome Biol. 2015 Dec 1;16:264. doi: 10.1186/s13059-015-0832-9 (PMC4665721; doi:10.1186/s13059-015-0832-9)

Supplementary Fig. 1

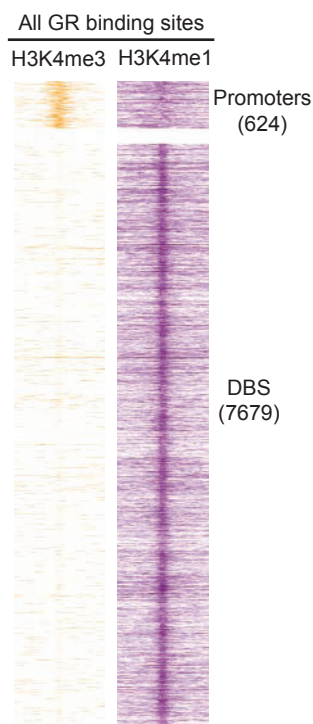

Supplement: Additional file 1: Figure S1. — GR binds predominantly to distal binding sites. Pile-up heat map depicting the H3K4me1 and H3K4me3 signal around (±12 kb) all GR-bound promoters and DBSs. (PDF 272 kb) [file 13059_2015_832_MOESM1_ESM.pdf]

Supplementary Fig. 2

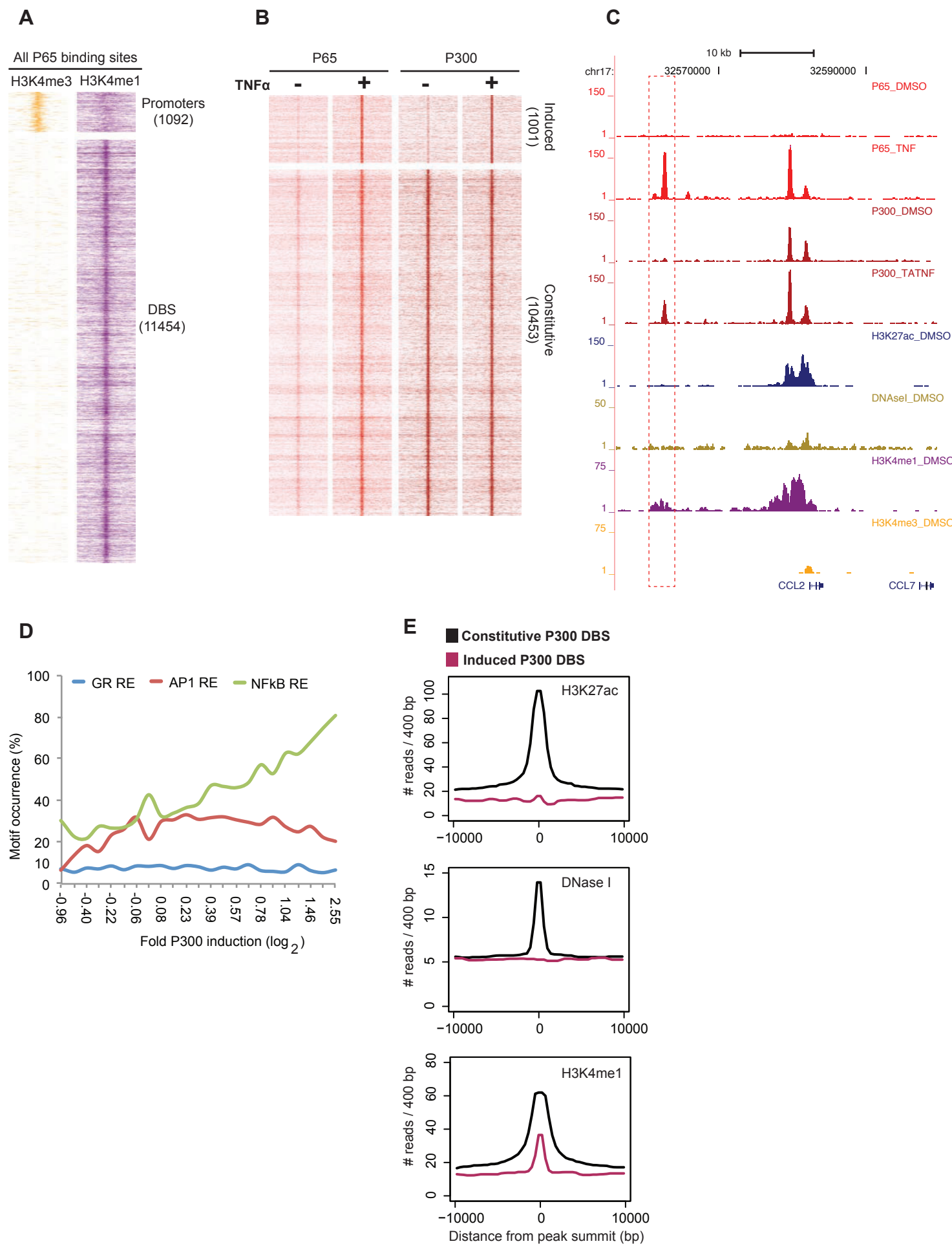

Supplement: Additional file 2: Figure S2. — Activated p65 induces de novo P300 depositions to latent genomic loci. (A) Pile-up heat map depicting the H3K4me1 and H3K4me3 signal around (±12 kb) all p65-bound promoters and DBSs. (B) Pile-up heat map depicting the p65 and P300 signal at all p65-bound enhancers upon vehicle, DMSO (−), and TNFα (+) treatment. (C) Example screenshot depicting TNFα-induced P300 recruitment at genomic regions (red box) and recruitment of p65 at genomic loci that are pre-marked by P300. (D) Motif occurrence at all p65-bound DBS presented as a function of TNFα-dependent P300 recruitment (x-axis) (top-panel). Level of shared binding of p65 and other TFs at all p65-bound DBS, presented as a function of TNFα-dependent P300 recruitment (bottom panel). (E) Level of H3K27ac, DNase I hypersensitivity, and H3K4me1 at all p65-bound DBSs (induced and constitutive P300 sites). (PDF 909 kb) [file 13059_2015_832_MOESM2_ESM.pdf]

Supplementary Fig. 3

**A**

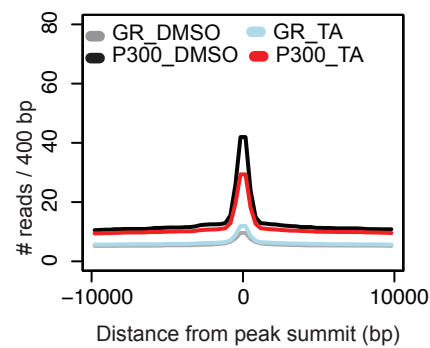

**B**

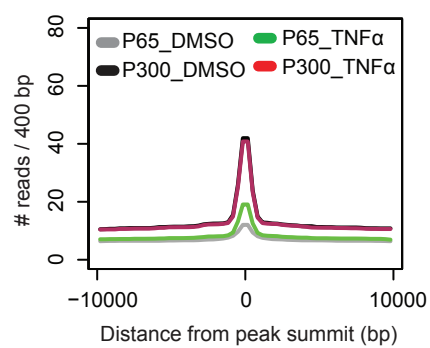

Supplement: Additional file 3: Figure S3. — Large numbers of P300-bound loci are not co-occupied by GR or p65. (A) Average ChIP-seq signal of GR and P300 around (±10 kb) all the P300 binding sites that do not show a significant GR occupancy. (B) Average ChIP-seq signal of p65 and P300 around (±10 kb) all the P300 binding sites that do not show a significant p65 occupancy. (PDF 168 kb) [file 13059_2015_832_MOESM3_ESM.pdf]

Supplementary Fig. 4

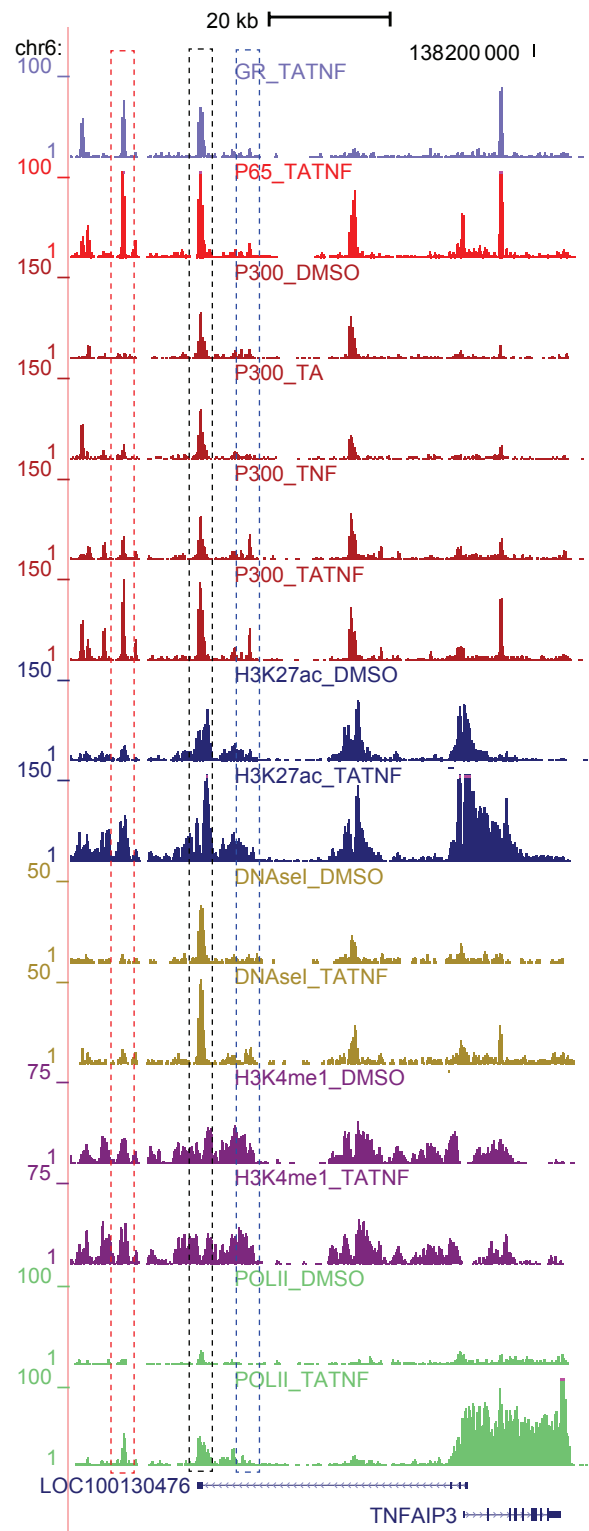

Supplement: Additional file 4: Figure S4. — Complex epigenetic changes induced by co-activated GR and p65. An example screenshot depicting the GR-dependent (blue box), p65-dependent (red box), and co-stimulation-dependent (black box) induced P300 DBSs and constitutive DBSs. Dynamic changes in DNase I accessibility, epigenetic modifications, and RNA-POLII activity on genes upon co-activation are noticeable characteristics of this locus. (PDF 236 kb) [file 13059_2015_832_MOESM4_ESM.pdf]

Supplementary Fig. 5

A

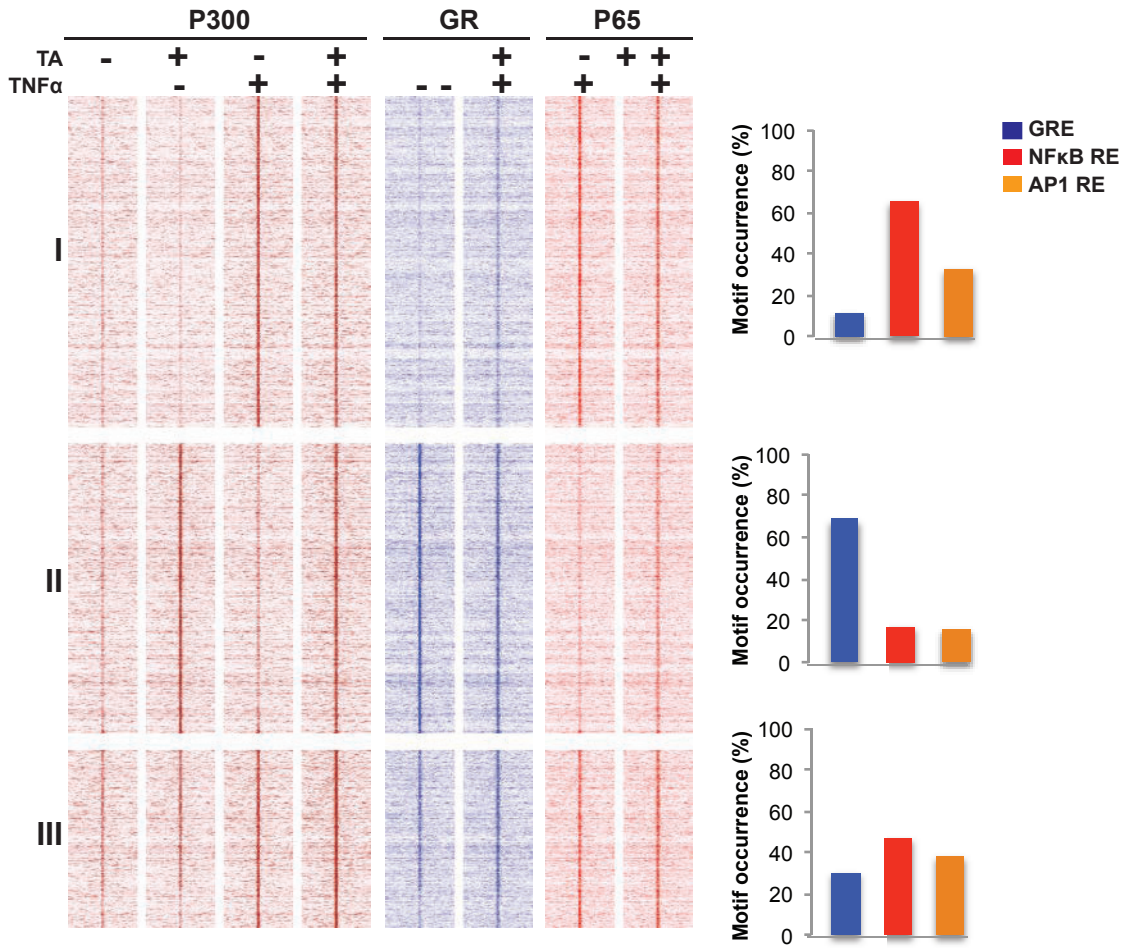

B

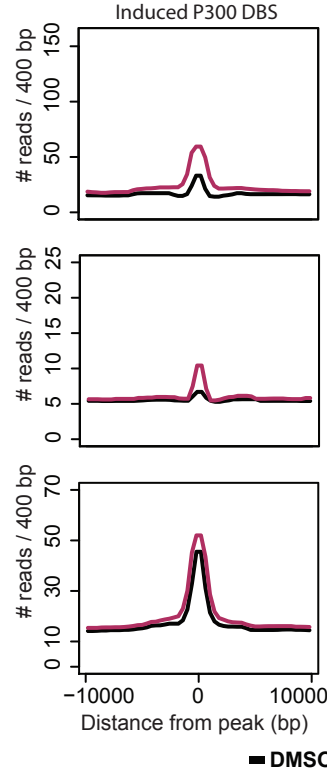

C

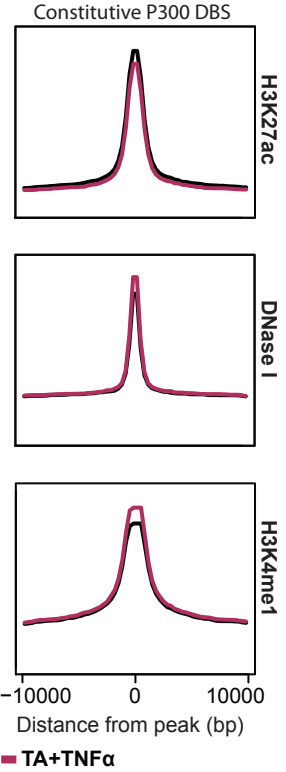

Supplement: Additional file 5: Figure S5. — Co-activation of GR and p65 induces additional de novo P300 DBSs. (A) Pile-up heat map depicting signal of P300, GR, and p65 at three groups (p65 dependent, GR dependent, and co-stimulation dependent) of inducible P300 DBSs that were identified upon co-activation of GR and p65. Motif occurrence (%) at all three clusters of induced P300 DBSs (bar graphs). (B) H3K27ac, DNase I accessibility, and H3K4me1 signal at all induced P300 DBSs upon vehicle (DMSO) and co-treatments (TA + TNFα). (C) H3K27ac, DNase I accessibility, and H3K4me1 signal at all constitutive P300 DBSs upon vehicle (DMSO) and co-treatments (TA + TNFα). (PDF 1137 kb) [file 13059_2015_832_MOESM5_ESM.pdf]

Supplementary Fig. 6

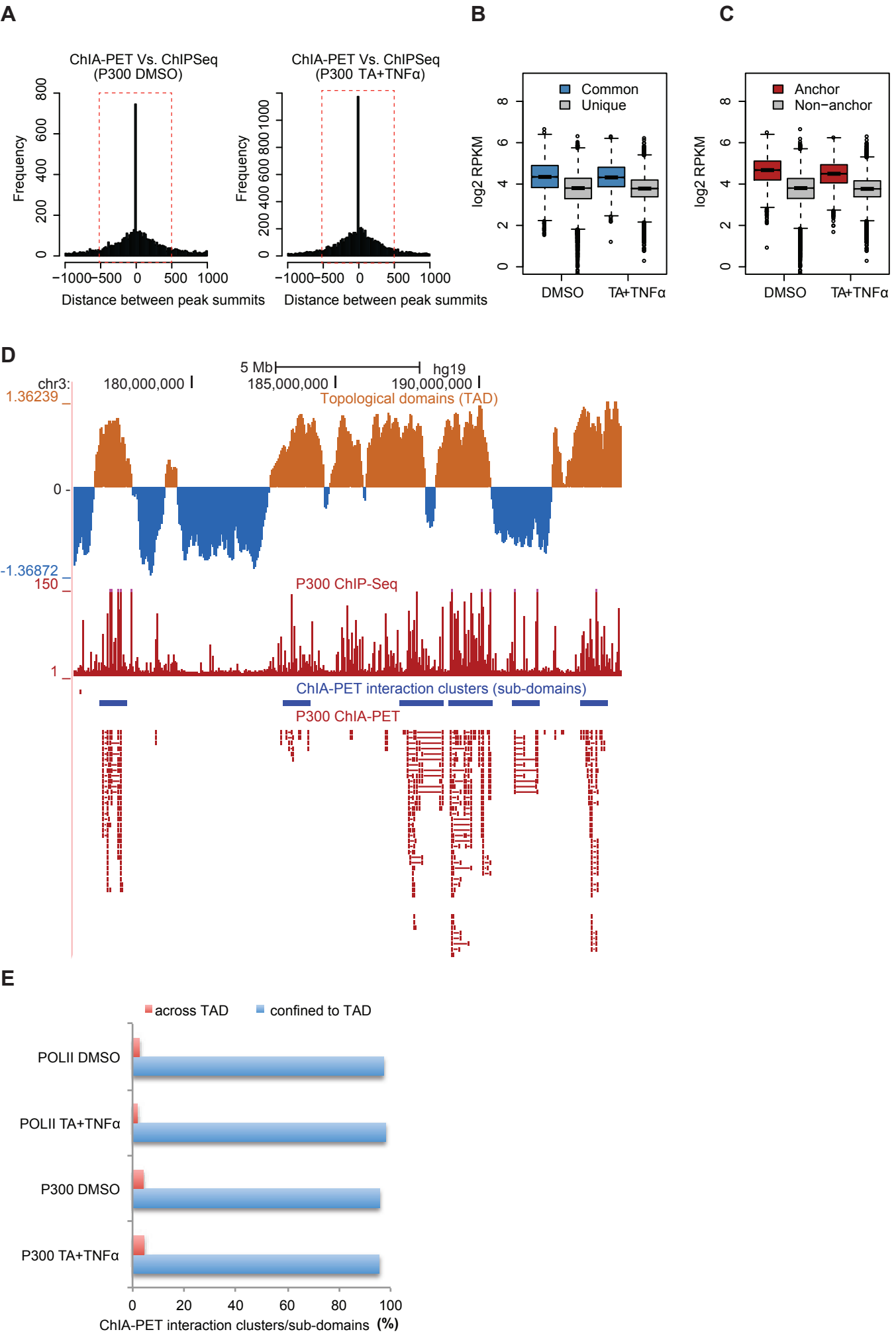

Supplement: Additional file 8: Figure S6. — (A) Histogram depicting the genomic proximity (localization) of P300 binding sites identified by ChIP-seq in relation to those identified by using ChIA-PET self-ligation PETs. Identical comparison is performed for both DMSO-treated and TA + TNFα-treated data sets. (B) P300 ChIP-seq signal at P300 binding sites commonly identified by ChIP-seq and ChIA-PET and those binding sites that were uniquely detected in the ChIP-seq data set. (C) P300 ChIP-seq signal at P300 binding sites that were either involved (anchor) or not involved (non-anchor) in long-range interaction as identified by ChIA-PET analysis. (D) An example screenshot depicting the P300 interaction subdomains, P300 ChIP-seq binding sites in relation to topological domains as defined by replication timing data (www.replicationdomain.org). (E) Localization of all the interaction subdomains identified by ChIA-PET analysis (P300 and POLII) in relation to topological domains. (PDF 1041 kb) [file 13059_2015_832_MOESM8_ESM.pdf]

Supplementary Fig. 7

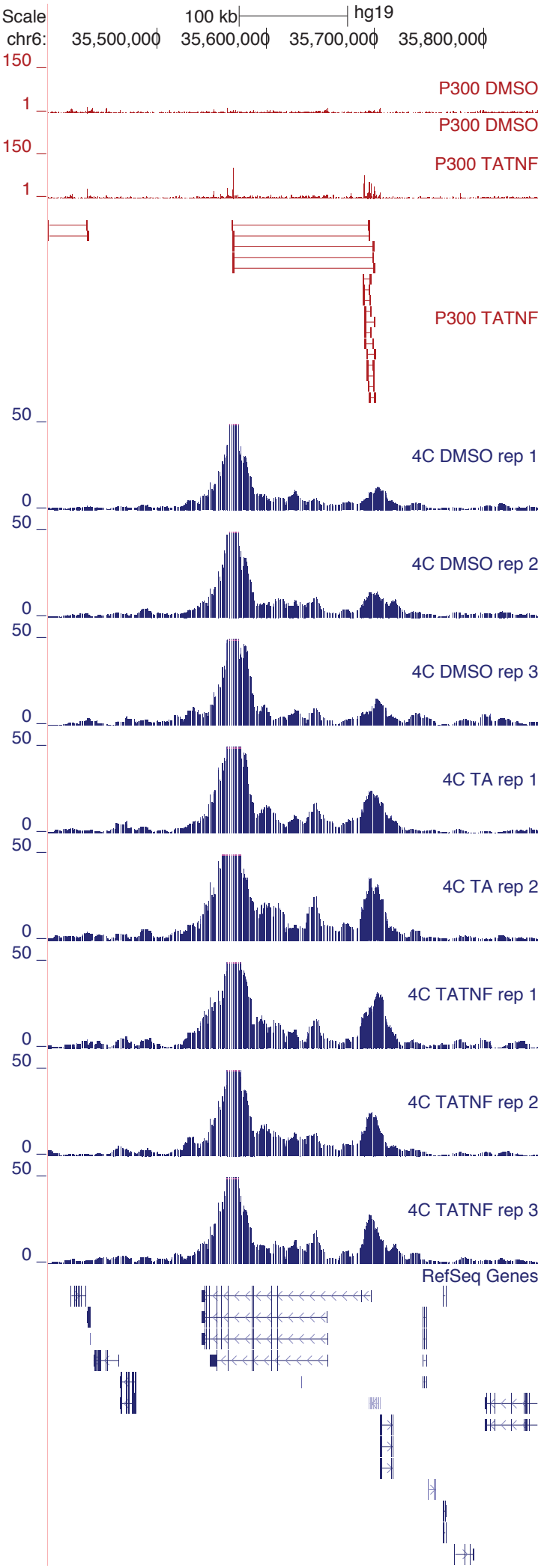

Supplement: Additional file 10: Figure S7. — Reproducibility of 4C-seq biological replicates at the FKBP5 locus. (PDF 1094 kb) [file 13059_2015_832_MOESM10_ESM.pdf]

### Supplementary Fig. 8

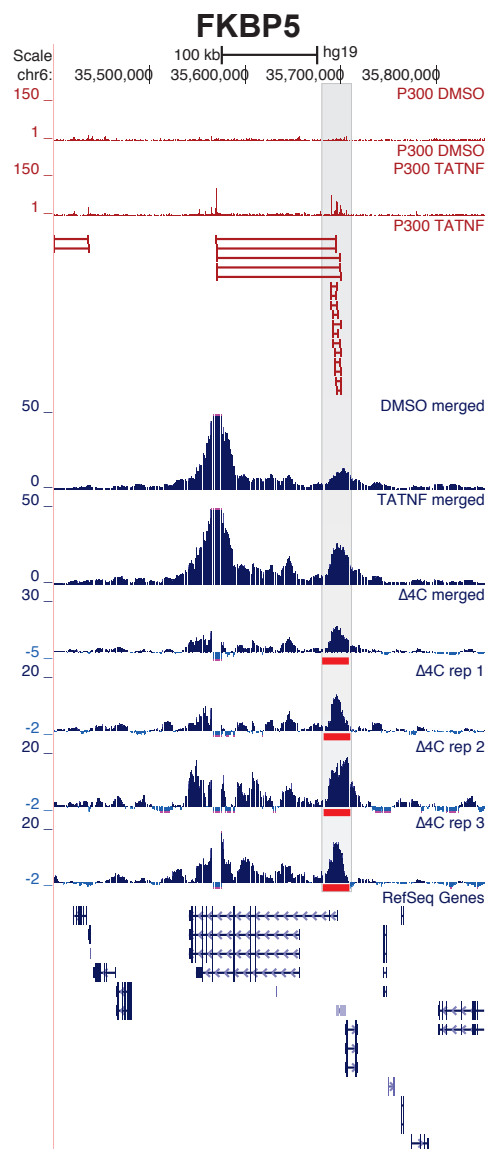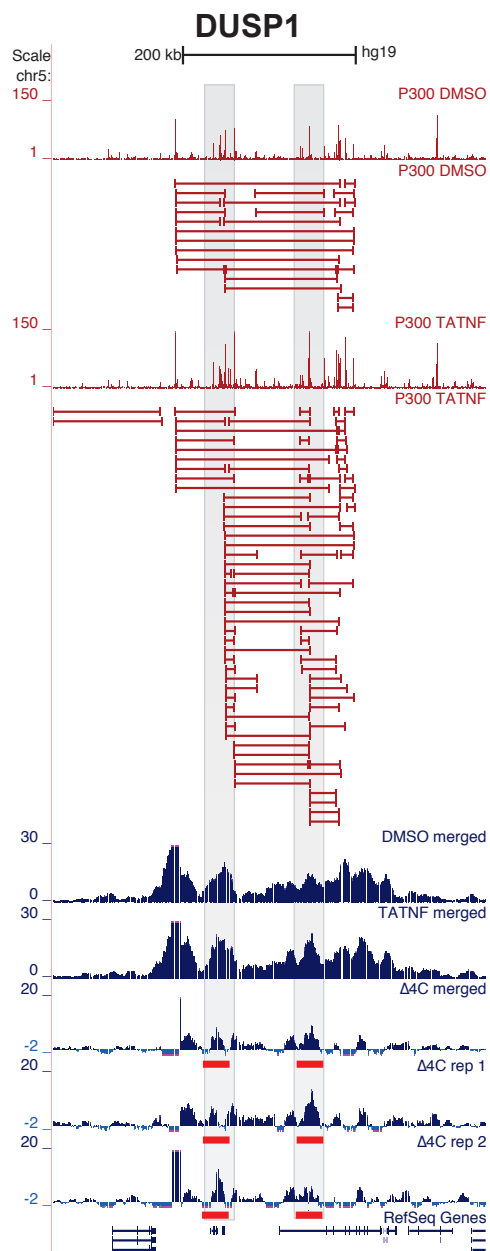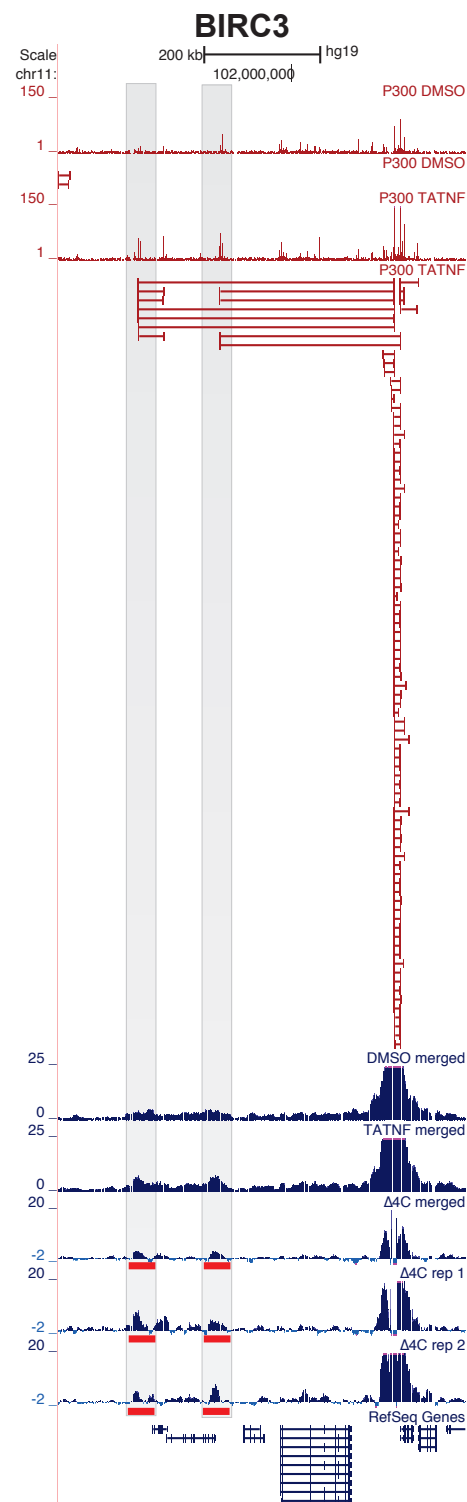

Supplement: Additional file 11: Figure S8. — Direct comparison of long-range interactions identified by P300 ChIA-PET and 4C-seq analyses at FKBP5, DUSP1, and BIRC3 loci. (PDF 1037 kb) [file 13059_2015_832_MOESM11_ESM.pdf]

Supplementary Fig. 9

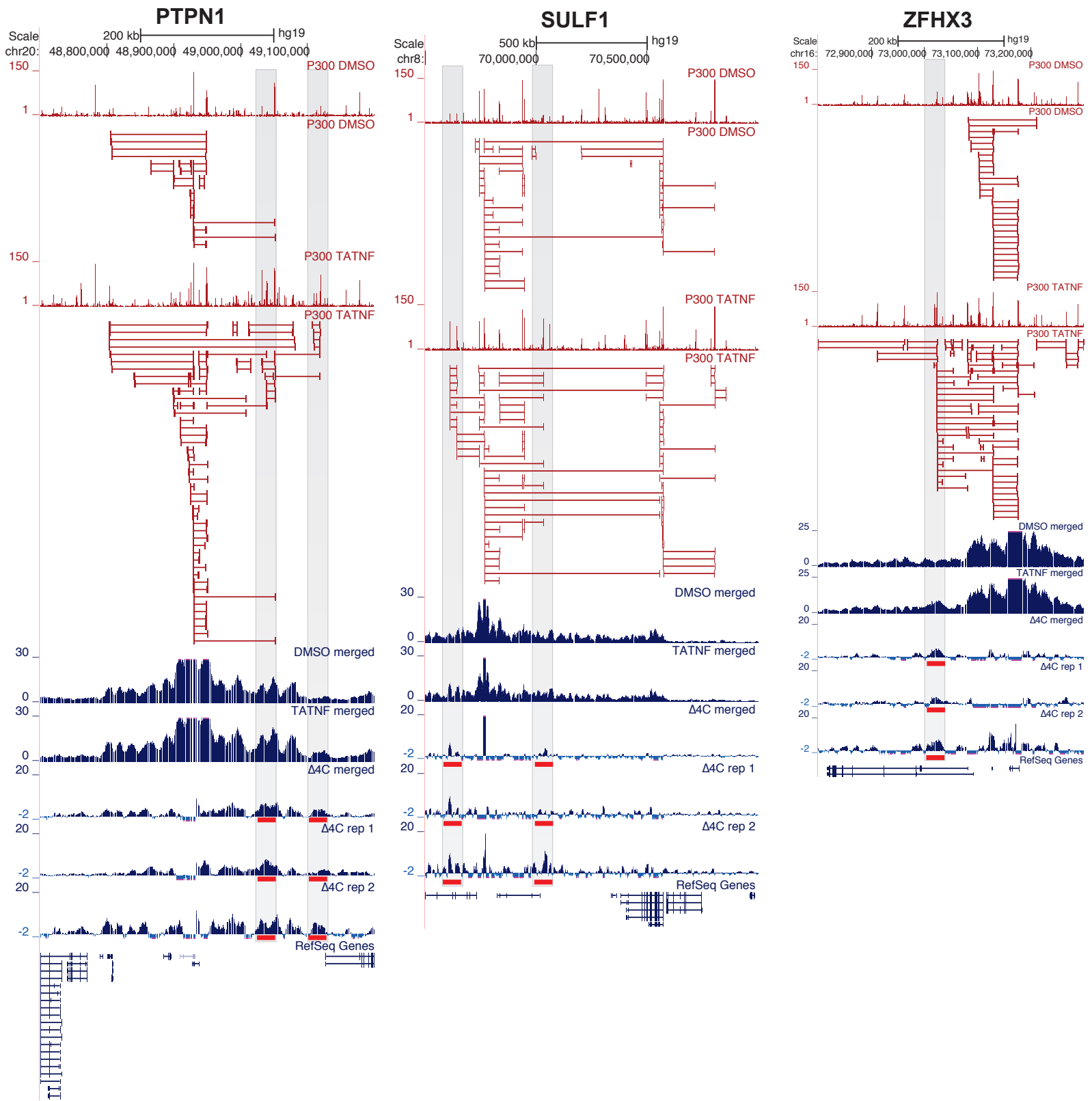

Supplement: Additional file 12: Figure S9. — Direct comparison of long-range interactions identified by P300 ChIA-PET and 4C-seq analyses at PTPN1, SULF1 and ZFHX3 loci. (PDF 1147 kb) [file 13059_2015_832_MOESM12_ESM.pdf]
