# Supplementary material for: Glucocorticoid receptor and nuclear factor kappa-b affect three-dimensional chromatin organization
Source: Genome Biol. 2015 Dec 1;16:264. doi: 10.1186/s13059-015-0832-9 (PMC4665721; doi:10.1186/s13059-015-0832-9)
Supplement: Additional file 6: — Supplementary text and Supplementary references. Cross-talk between GR and NFκB leads to complex changes in chromatin landscape. (DOCX 73 kb) [file 13059_2015_832_MOESM6_ESM.docx]

**SUPPLEMENTAL NOTE**

**Title: Glucocorticoid receptor and nuclear factor kappa-b affect 3D chromatin organization.**

Tatyana Kuznetsova^1,4^, Shuang-Yin Wang^1,4^, Nagesha A. Rao^1,4^, Amit Mandoli^1^, Joost Martens^1^, Nils Rother^1^, Aafke Aartse^1^, Laszlo Groh^1^, Eva M. Janssen-Megens^1^, Guoliang Li^3^, Yijun Ruan^2^, Colin Logie^1^, Hendrik G. Stunnenberg^1,5^

^1^Radboud University, Department of Molecular Biology, Faculty of Science Nijmegen, the Netherlands.

^2^The Jackson Laboratory for Genomic Medicine, and Department of Genetic and Development Biology, University of Connecticut, 400 Farmington, CT 06030, USA.

^3^National Key Laboratory of Crop Genetic Improvement, College of Informatics, Huazhong Agricultural University, China.

^4^ These authors contributed equally to this work.

^5^ Corresponding author

E-MAIL: h.stunnenberg@ncmls.ru.nl; TEL: +31-24-3610524; FAX: +31-24-3610520

**SUPPLEMENTARY TEXT/REFERENCES**

**Cross-talk between GR and NFkB leads to complex changes in chromatin landscape**

We [1] previously reported a staggering complexity in the cross talk between the GR and P65 pathways with positive and negative effects of co-stimulation with respect to gene expression, DNA binding patterns of p65 and GR, and consequently for epigenetic makeup. An illustration of this complexity is exemplified by the TNFAIP3 locus (Supplemental Fig. 4). TNFAIP3 is a bona fide target gene of NFκB as well as GR that synergistic responds to co-activation by glucocorticoids and TNFα [1-3].

To gain global insight, we co-activated GR and p65 and assessed P300 occupancy, epigenetic marking and DNaseI accessibility. Co-activation leads to *de novo* recruitment of P300 to 2884 DBS as compared to 1911 sites when adding up the induced P300 binding site obtained after single activation (2 x median absolute deviation). K-means clustering of these DBS identified three distinct groups. At the vast majority of induced P300 sites detected following single TNFα activation (Supplemental Fig. 2B, C), additional activation and binding of GR did not alter the P300 occupancy (Supplemental Fig. 5A, I). GR appears to join a DBS that is activated by p65 in response to TNFα treatment. Similarly, TNFα co-stimulation and binding of p65 did not notably alter the P300 inducible sites that were established upon single activation of GR (Supplemental Fig. 5A, II). At an additional ~700 DBS (Supplemental Fig. 5A, III), co-activation of GR and p65 led to a modest synergistic increase in P300 recruitment. Taken together, we identify induced P300 DBS that are uncovered upon single activation of GR, P65 and their co-activation.

We also analyzed changes in DNase I accessibility and epigenetic marks at all induced P300 DBS (Supplemental Fig. 5B) upon co-activation of GR and P65 and compared with that of constitutive P300 DBS (Supplemental Fig. 5C). Induced P300 DBS display a readily detectable gain in DNase I accessibility and H3K27ac upon co-activation of GR and P65 (Supplemental Fig. 5B, two upper panels). These loci are marked with H3K4me1 in un-stimulated cells, marginally gain H3K4me1 signal upon co-stimulation (Supplemental Fig. 5B, lower panel). On contrary, constitutive P300 DBS that are DNase I hypersensitive, marked with H3K27ac and H3K4me1 prior to ligand/s stimulation, show no further changes upon to co-stimulation (Supplemental Fig. 5C). Finally, P300 occupancy is also marginally reduced at a small subset of DBS (~400) upon co-stimulations (data not shown).

**Supplementary references**

1. Rao NAS, McCalman MT, Moulos P, Francoijs K-J, Chatziioannou A, Kolisis FN, Alexis MN, Mitsiou DJ, Stunnenberg HG (2011) Coactivation of GR and NFKB alters the repertoire of their binding sites and target genes. Genome Research 21:1404–1416

2. Krikos A, Laherty CD, Dixit VM (1992) Transcriptional activation of the tumor necrosis factor alpha-inducible zinc finger protein, A20, is mediated by kappa B elements. Journal of Biological Chemistry 267:17971–17976

3. Altonsy MO, Sasse SK, Phang TL, Gerber AN (2014) Context-dependent cooperation between nuclear factor κB (NF-κB) and the glucocorticoid receptor at a TNFAIP3 intronic enhancer: a mechanism to maintain negative feedback control of inflammation. J Biol Chem 289:8231–8239
